# Assessing Gait Stability before and after Cochlear Implantation

**DOI:** 10.1155/2019/2474273

**Published:** 2019-01-14

**Authors:** Katarzyna Kaczmarczyk, Michalina Błażkiewicz, Ida Wiszomirska, Katarzyna Pietrasik, Agnieszka Zdrodowska, Andrzej Wit, Gabor Barton, Henryk Skarżyński

**Affiliations:** ^1^Department of Physiology, Faculty of Rehabilitation, Józef Piłsudski University of Physical Education in Warsaw, Poland; ^2^Department of Biomechanics, Faculty of Rehabilitation, Józef Piłsudski University of Physical Education in Warsaw, Poland; ^3^Department of Anatomy and Kinesiology, Faculty of Rehabilitation, Józef Piłsudski University of Physical Education in Warsaw, Poland; ^4^Otorhinolaryngology Surgery Clinic, Institute of Physiology and Pathology of Hearing, Warsaw, Poland; ^5^World Hearing Center, Institute of Physiology and Pathology of Hearing, Warsaw, Kajetany, Poland; ^6^Department of Physical Therapy and Massage, Faculty of Rehabilitation, Józef Piłsudski University of Physical Education in Warsaw, Poland; ^7^Research Institute for Sport and Exercise Sciences, Liverpool John Moores University, UK

## Abstract

**Background:**

It is known that cochlear implantation may alter the inner ear and induce vestibular disorders.

**Research Question:**

How does cochlear implantation influence gait stability?* Material and Methods*. An experimental group of twenty-one subjects scheduled for cochlear implantation underwent gait testing twice, on the day before cochlear implantation (BCI) and three months after cochlear implantation (ACI), using a motion capture system. A control group of 30 age-matched healthy individuals were also tested.

**Results:**

In the experimental group, the gait stability ratio (GSR) was found to improve in 17 subjects after implantation, by an average of 6%. Certain other parameters also showed statistically significant improvement between the two experimental group tests: step time (p<0.001), single-support phase walking speed (p<0.05), and center of mass (CoM) (p<0.05). Using the CoM results of the control group, we devised a stability classification system and applied it to the pre- and postimplantation subjects. After implantation, increases were seen in the number of subjects classified in interval II (strong stability) and III (weak stability). The number of subjects in interval I (perfect stability) decreased by 1 and in interval IV (no stability) by 4.

**Significance:**

(1) Although cochlear implantation intervenes in the vestibular area, we found evidence that gait stability improves in most subjects after the surgery, reducing the risk of falls. (2) We found statistically significant improvements in individual parameters (such as single-support phase time), in GSR, and in CoM. (3) Based on CoM results, we proposed a new rule-of-thumb way of classifying patients into gait stability intervals, for use in rehabilitation planning and monitoring.

## 1. Background

Sensorineural hearing loss is the third leading cause of disability in adults worldwide [1]. To restore their hearing ability and improve their quality of life, some people with profound hearing loss may undergo cochlear implantation, a surgical procedure that involves inserting a multielectrode array directly into the cochlea. Due to the anatomical proximity between the vestibular system and the cochlea, however, such surgery can unfortunately lead to vestibular damage. The procedure of insertion may induce vestibular disorders after the surgery itself, or after the activation of the cochlear implant [2]. The risk of lost vestibular function may depend in part on the particular surgical method used [3]; the technique of accessing the inner ear through a round window has proved to be an extremely effective method in treating partial deafness [4–6]. The incidence of damage to vestibular function and postoperative complications has been estimated variously in the available literature, from only sporadic cases up to 75% of all adult patients [7–10]. The most common symptoms are vertigo, dizziness, and imbalance [10]. The risk of falls has been found to increase in 56% of all cochlear implantees (and 40% of implantees who had normal balance prior to implantation); older implantees have also been found to exhibit worse balance than younger implantees [11–13]. Most of these complications are episodic, and some are transient [14], but researchers also point to delayed effects and possible histopathologic damage that may not present symptoms [13, 15–17].

However, despite such possible damage and complications, evidence of improved postural stability after cochlear implantation has also been reported [18, 19]. Possible reasons for this include compensation of a previously uncompensated vestibular lesion [19] and improved spatial orientation resulting from recovered hearing [20, 21]. In such cases, auditory recovery may be provoking motor learning and the development of new neural networks, which in turn could lead to improved postural stability [10].

The vestibular system is, of course, just one source of information contributing to balance control, alongside visual, somatosensory, and even auditory input [22, 23]. Various studies have considered disorders of postural stability in patients with hearing loss, as well as the impact of cochlear implantation on postural stability [10, 19, 24]. However, the majority of falls occur during ambulation, not static stance, and a key factor in preventing falls is the ability to quickly take an appropriate recovery step. This suggests that certain post-CI complications, such as heightened risk of falling, may be better reflected by measuring gait stability, rather than postural stability. Nevertheless, there have been no studies evaluating how cochlear implantation relates to gait stability. On the other hand, the complex and diverse etiology of balance problems makes them a difficult diagnostic and treatment issue [25]—although several methods for quantifying gait stability have been presented in the literature [26–30], they have been proposed as a tool for predicting the risk of falls in the elderly and have not been applied to the study of gait stability in patients after cochlear implantation.

The aim of our study, therefore, was to report a first-ever assessment of gait stability in patients before and after cochlear implantation (using two measures of gait stability: the *β*-coefficient and the gait stability ratio) and to propose a stability classification system based on the movement of the center of body mass (CoM) in the transverse plane.

## 2. Material and Methods

### 2.1. Participants

Subjects (n=21) for the experimental group were recruited from the World Hearing Center, Institute of Physiology and Pathology of Hearing, Warsaw, Kajetany, Poland. Those qualified for the experimental group were adults identified as partial deafness (PD) patients, having normal low-frequency hearing but no hearing in the high-frequency range [4], scheduled for cochlear implantation (CI). CI is received by patients who find that conventional hearing aids do not bring sufficient benefits; they are qualified for the procedure by a special commission, after a series of specialist tests and consultations. Exclusion criteria include any significant presurgery psychological barriers, aversion to implantation, or contraindications to receiving anesthesia during surgery. The implantation was performed on the right side in 13 subjects, on the left in 8. The CI was inserted unilaterally using the round window surgical technique in 18 subjects, via the cochleostomy procedure in the 3 remaining subjects (for whom the round window technique was deemed inappropriate). No participants in the experimental group reported any sensory impairment or physical injury that hindered the performance of balance trials, nor did any of the participants have previous experience with balance training.

These subjects were compared against a control group of 30 healthy subjects of similar age, recruited among students applying for classes at the University of the Third Age in Warsaw. No participants from this control group reported any sensory impairment or physical injury that hindered performance of the balance task, nor did any of the participants have previous experience with balance training.

Participants were qualified for both groups by a physician based on an interview, a clinical exam, vision damage evaluation, EKG, an exam of their hearing, the condition of their cranial nerves, any potential meningitis symptoms, and cerebellar tests (the nose-finger test, diadochokinesis, deflection test, and static-dynamic stance and gait tests—the Romberg test, Unterberger test, Babiński-Weil test, Fukuda test, and straight march test). Moreover, in the experimental group, pre- and postoperative vestibular function were tested as follows: videonystagmography (VNG), vestibular evoked myogenic potentials (VEMP), the video head impulse test (vHIT), and computerized dynamic posturography sensory organization test (CDP-SOT). All subjects gave informed written consent to the experimental procedures, which were approved by the local ethics committee. Demographics for the experimental and control groups are presented in Table 1.

### 2.2. Instrumentation and Data Collection

The experimental group was tested in two stages: one on the day before surgery (BCI—before cochlear implantation) and the other three months after implantation (ACI—after cochlear implantation). In the first case participants were not using hearing aids, and in the second their cochlear implant was turned off. The control group, in turn, was tested once.

Gait studies were conducted using a motion capture system (Vicon Motion Systems Ltd, Oxford, UK). First, anthropometric measurements were taken for each person. Next, spherical markers were placed at anatomical landmarks, according to the standards of the biomechanical model Plug-In-Gait available within the Vicon system. A motion capture system, consisting of nine infrared cameras, was employed to collect kinematics data at a sampling rate of 100 Hz. The system was precalibrated according to the manufacturer's recommendations. Each subject performed three trials of unassisted walking at their preferred walking speed along a 10m walkway. For each individual, one trial, performed naturally and without any random mistakes (without recording errors or problems with markers, without the subject stopping for some reason, failing to reach the end of the path, etc.) was selected and taken into account for further analysis.

### 2.3. Determination of Gait Stability

We evaluated two measures of gait stability: the gait stability ratio (GSR), as a more generic measure of stability, and the *β*-coefficient, which measures straightness of gait, i.e., the ability to maintain a steady direction of motion (without deviating to the left or right).

#### 2.3.1. Spatiotemporal Parameters

The following spatiotemporal parameters were exported from the Vicon Nexus software: gait speed, cadence, stride and step time, stride and step length, step width, timing of single support, and double support. The gait stability ratio (GSR) was calculated based on the cadence and velocity measures using the following equation: GSR = cadence/velocity [31]. In this equation, the GSR units are steps per meter. The ratio represents a measure of walking stability and provides a mechanism for normalizing cadence with respect to velocity. Therefore, as GSR increases, participants are taking more steps within a 1 m distance and spending a greater proportion of the walking cycle in contact with the floor. By shortening the duration of single-support and therefore increasing the duration of double support, participants increase their gait stability.

In order to determine the range of improvement of gait stability in individual subjects, the ΔGSR difference was calculated as Δ*GSR* = *GSR*_*BCI*_ − *GSR*_*ACI*_, where GSR_BCI_ represents preimplantation and GSR_ACI_ postimplantation data. Positive ΔGSR values indicate that the gait became more stable, with subjects spending less time in the double-support phase.

#### 2.3.2. CoM Determination

**Figure 1:**
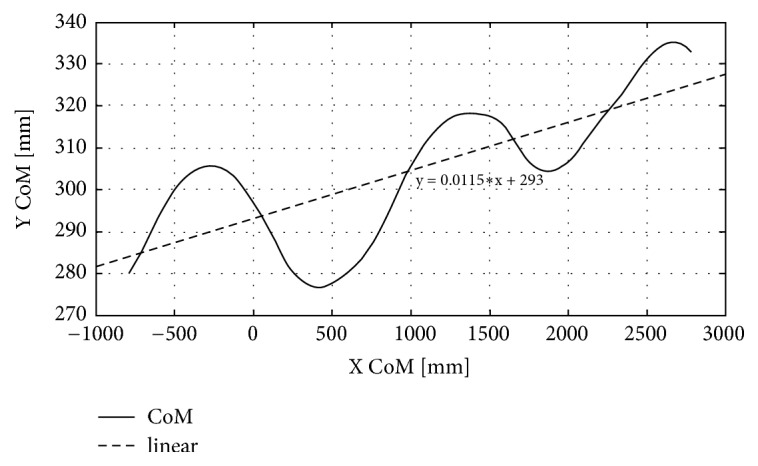
Sample graph representing shifting the center of body mass (CoM) in free gait in the transverse plane.

In trials where three gait cycles were recorded, the movement of the CoM in the transverse plane was taken into consideration. Although no specific instructions were given to the subjects, the 10 m long walkway provided visual cues which guided them to walk in the center line of the walkway. After the curves were plotted (Figure 1), a linear trend curve was drawn for each one with the equation* y = ax + b*. The coefficient of the variable x is equal to the tangent of the slope angle *β* relative to the x-axis: *a* = *tg*(*β*). Therefore, the angle (measured in degrees) at which the CoM trajectory deviates from the forwards direction can be calculated as *β* = *arctg*(*a*).

The results of the control group were used to establish a set of stability intervals for classification purposes. The intervals were created based on standard deviations for the *β*-coefficient, calculated as arctg(a). Stable gait may be defined based on the *β*-coefficient as -1°<*β*< 1° (with positive values indicating instability in one direction, negative values the other direction). However, as no negative *β*-coefficient was recorded for any individual in the study, for our purposes we defined stable gait as 0°<*β*< 1°.

### 2.4. Statistical Analysis

First, in order to assess the normal distribution for all parameters in the three groups, the Shapiro–Wilk test was used. All data were analyzed at a significance level of *α* = 0.05 using Statistica 12.0 (StatSoft, PL). Next, three comparisons were made as follows: (1) an intragroup comparison between BCI and ACI and (2) two intergroup comparisons, between BCI and controls and between ACI and controls. In the case of the BCI vs. ACI comparison the Wilcoxon signed-rank test was used. In case of ACI vs. C and BCI vs. C the U-Mann–Whitney test was used.

## 3. Results

### 3.1. Spatiotemporal Parameters

For the control group (C), deviations in distribution were found for the variables GSR (p = 0.0276) and CoM (p = 0.0201). For the preintervention group (BCI), deviations in distribution were found for cadence (p = 0.0013), stride and step time (p = 0.0070 and p = 0.0027), and walking speed (p = 0.0013). For the postimplantation group (ACI), on the other hand, all parameters tested had normal distribution apart from double-support phase (p = 0.0003).

### 3.2. Intragroup Comparison between BCI and ACI

The Wilcoxon signed-rank test was applied to the following spatiotemporal parameters: cadence, stride and step time, timing of single support and double support, step width, stride and step length, walking speed, CoM, and GSR. The Wilcoxon signed-rank test was highly significant for six variables: step time (p = 0.0001), single-support phase (p = 0.0001), walking speed and cadence (p = 0.0227), CoM (p = 0.0117), and GSR (p = 0.0001). Thus, we can conclude that these variables differed significantly between the two experimental groups (BCI, ACI).

**Figure 2:**
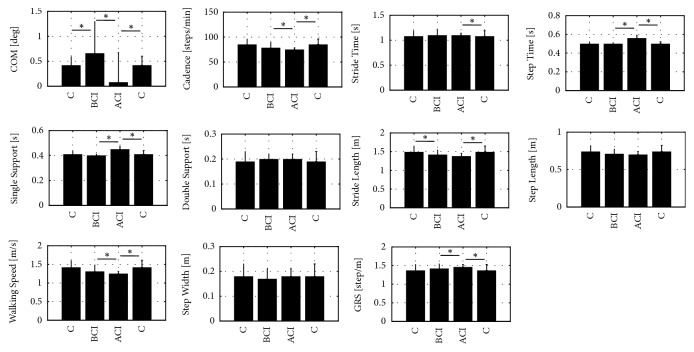
Results for **A**. Wilcoxon signed-rank test between BCI and ACI group and **B**. U-Mann–Whitney test between BCI vs. C and ACI vs. C groups.

The detailed results, given in Figure 2, show a significant difference for six of the 11 variables between the BCI and ACI groups, whereas there were statistically significant differences between the ACI and C groups.

### 3.3. Two Intergroup Comparisons: BCI vs. C and ACI vs. C

The U-Mann–Whitney test was used to compare both the preimplantation results (BCI) and the postimplantation results (ACI) to the control group (C).

The BCI vs. C comparison showed statistically significant differences only for stride length (p = 0.0465) and CoM (p = 0.0216). The stride length values were greater for the control group, whereas CoM fluctuations were smaller for the control group (Figure 2).

The ACI vs. C comparison, in turn, found statistically significant differences for eight parameters: CoM fluctuations, cadence, stride and step length, walking speed, and step width showed significantly greater values in the control group, and the other two parameters in the ACI group.

As noted above, information about gait stability can also be gleaned from the GSR parameter.

**Figure 3:**
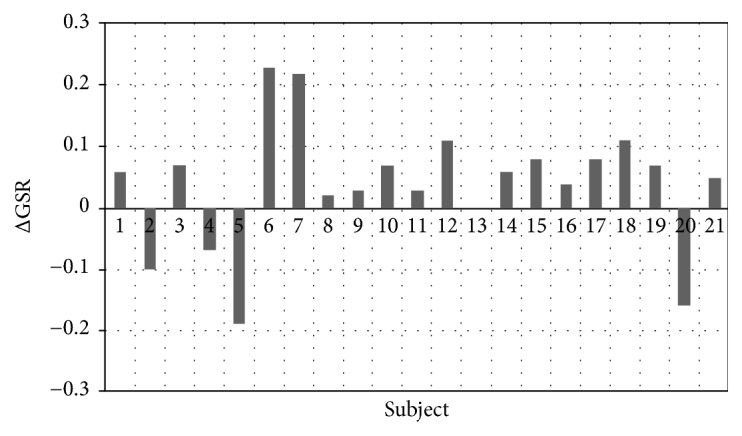
ΔGSR values for individual subjects.

Figure 3 presents the differences in GSR before and after cochlear implantation (ΔGSR), showing that 17 subjects improved, by an average of 6%, whereas only 4 did not.

### 3.4. CoM Determination

As a useful way of classifying gait stability, we devised four intervals based on the results of healthy subjects (Table 2). Stable gait was defined as -1°<*β*< 1°. If the angle was *β*< 0 the subject showed leftward movement, while *β*> 0 indicated rightward movement.

**Figure 4:**
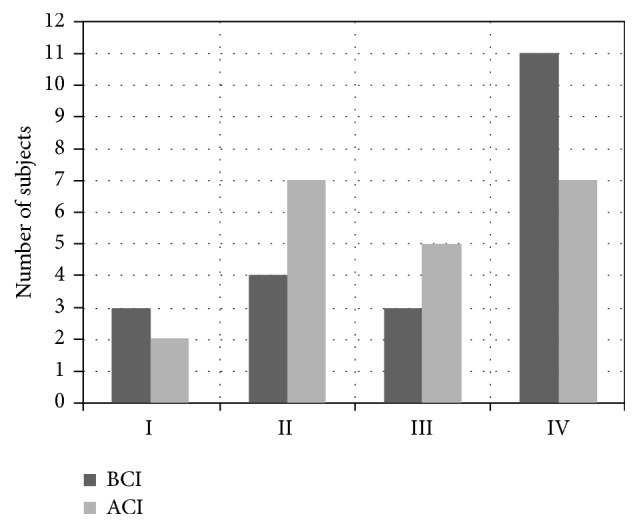
Number of subjects in each interval before and after cochlear implantation.

Figure 4 shows the number of subjects before and after implantation who, depending on the direction of CoM movement, were classified in intervals I, II, III, or IV.

After implantation, the number of subjects who exhibited perfect stability (interval I) decreased by 1. The number of subjects in interval IV (no stability) also decreased by 4. Increases were seen in the number of subjects classified in interval II (strong stability) and III (weak stability).

## 4. Discussion

Cochlear implantation (CI) may induce vestibular impairment, which may reduce one source of input to balance control mechanisms and therefore contribute to dizziness, vertigo, and imbalance. These impairments most often occur soon after surgery (acute symptoms of vestibular impairment being limited to the first month postsurgery), as well as after implant activation [2, 32] but implantation may also cause significant, though possibly asymptomatic, histopathologic damage of the vestibular end organs [16, 17]. Our study focused on the longer-term consequences of vestibular effects on gait, with the average postimplantation time for our group being 3.2 months.

Published reports describing the effect of a cochlear implant on stability provide conflicting conclusions, ranging from observed negative effects [15] to improved postsurgery stability in static and dynamic studies [19, 32]. The causes for this lack of consistency may include differences in research methodology, nonstandardized testing methods, nonhomogeneous groups of subjects, and applying a short-term rather than long-term approach, as well as a lack of presurgery measurements [2, 3, 8].

Moreover, Meheu et al. [27] recently reported that vestibular status prior to CI is a major predictor of post-CI postural control. Their study showed that only those participants who had unilateral vestibular abnormality and who received cochlear implant in the ear with normal vestibular function showed postural differences after cochlear implantation. In our study, on the other hand, improvement was noted in most cases, but this may be a result of the fact that only patients without vestibular disturbances were qualified for participation.

The surgical technique of operating on the inner ear through a round window (RW) as well as reductions in the size of the implantable part of the cochlear implants has yielded improved effects of surgical intervention [4–6]. In our study group, more than 90% of subjects were operated on using the RW technique. However, Kluenter et al. [32] found no differences in postoperative vestibular and balance test results in patients undergoing standard cochleostomy (SC) vs. those treated with the RW technique. We therefore we did not separate patients into distinct SC (n=3) and RW (n=18) groups, but rather considered all pre- and postimplantation patients together.

Numerous studies have tried to assess imbalance based on gait parameter changes [31–36]. The studies by Cromwell and Newton [31] and Maki [33] found a strong correlation between impaired balance and gait in elderly patients. The most common parameters selected for evaluation were walking speed [34, 35], stride length [32], and double-support time [36]. Gait stability was found to decrease with any changes made to these parameters, such as decreased speed, shortened stride length, or longer double-support time [31]. However, as we noted above, there have as yet been no studies specifically addressing gait stability in patients before and after cochlear implantation.

Our measurements of the spatiotemporal gait parameters demonstrated improved gait stability in patients after cochlear implantation; as we noted in the Background section this may plausibly be due to an improved “spatial sense” resulting from auditory recovery, motoric learning, and the development of new neural networks. Spatiotemporal gait parameters have also been effectively used to assess individuals' adaptation to balance challenges [2, 33]. In studies comparing gait stability in the elderly and the young, Rogers et al. [35] found that spatiotemporal parameters (mainly velocity and cadence), measured separately, are not good predictors of dynamic balance. Their study found that the gait speed in their patients adapted under very challenging conditions, but not under low-challenge conditions. In this case, the GSR ratio proposed by Cromwell & Newton [31] provided a more sensitive measure of dynamic balance ability. In our study, more than 80% of subjects after cochlear implantation experienced improved gait stability, as gauged by the GSR ratio.

Gait stability assessment has attracted interest from many authors. Lee and Chou [29] demonstrated that the medial CoM-COP inclination angle is a sensitive measure of gait stability in the elderly. Similarly, Chou et al. [30] proved that linear measures of CoM motion in the frontal plane during obstacle crossing are an indicator of gait stability. Kaya et al. [37] used the body center of mass (CoM) and its relative position to the center of pressure (COP) of the supporting foot to examine gait stability. In turn, Hamacheret al. [26] argued that it is not CoM but the linear variability of temporal measures of swing and stance that is most important in gait stability assessment and capable of distinguishing between fallers and nonfallers. In our study, we reverted to the use of CoM to evaluate gait stability and proposed a new method of classifying gait stability based on direction of movement (straying to the left or right), delineating four evaluative intervals of gait stability on the basis of the results of healthy subjects. Before cochlear implantation surgery, the largest share of the subjects in our group fell into the worst interval (interval IV, “no stability”). This group also showed the largest improvement.

## 5. Conclusions

In short, we found that cochlear implantation does not impair gait stability, contrary to what might be expected given the surgical intervention to the vestibular system and given the postsurgical complications that are known to occur. Rather, we found that gait stability improved in most subjects after cochlear implantation, thereby reducing the risk of falling. In the experimental group, the gait stability ratio (GSR) improved in 17 subjects after cochlear implantation, by an average of 6%. Some individual parameters also showed statistically significant improvement between the two experimental group tests: step time (p<0.001), single-support phase walking speed (p<0.05), and CoM (p<0.05). We have speculated that the observed improvement in gait stability after cochlear implantation may plausibly be due to an improved “spatial sense” resulting from auditory recovery and to motoric learning.

Lastly, evaluating gait stability in terms of the angular deviation of the CoM trajectory from the forwards direction in the transverse plane, we devised a stability classification system based on the results of the control group and applied to the pre- and postimplantation subjects. After implantation, increases were seen in the number of subjects classified in intervals II (strong stability) and III (weak stability). The number of subjects in interval I (perfect stability) decreased by 1 and in interval IV (no stability) by 4.

## Figures and Tables

**Table 1:** The demographics for the experimental and control groups.

Groups	Age [yrs]	Body mass [kg]	Body height [m]	Gender	Etiology of deafness
Experimental	50.66±18.02	78.19±18.40	167.76±11.16	F=9	15 unclear
				M=12	2 after mumps
					2 meningitis
					1 congenital
					1 otosclerosis

Control	45.6±11.8	76.2±16.7	167.6±10.5	F=23	
				M=7	

**Table 2:** Agreement categorization for *β*–coefficient intervals.

Interval	*β* – coefficient intervals	Strength of agreement
I: $\left(\overset{-}{x}-SD,\overset{-}{x}+SD\right)$	(0.23°; 0,62°)	Perfect gait stability

II: $\left(\overset{-}{x}-2SD,\overset{-}{x}-SD\right)\cup \left(\overset{-}{x}+SD,\overset{-}{x}+2SD\right)$	(0.03°, 0.23°] ∪ [0.62°; 0.81°)	Strong gait stability

III: $\left(\overset{-}{x}-3SD,\overset{-}{x}-2SD\right)\cup \left(\overset{-}{x}+2SD,\overset{-}{x}+3SD\right)$	(-0.16°; 0.03°] ∪ [0.81°; 1°)	Weak gait stability

IV: $\left(-\infty ,\overset{-}{x}-3SD\right)\cup \left(\overset{-}{x}+3SD,+\infty \right)$	(-∞; -0.16°] ∪ [1°, +∞)	No gait stability

## Data Availability

The data used to support the findings of this study are restricted by the ethics committee in order to protect patient privacy. Data are available from Katarzyna Kaczmarczyk (katarzyna.kaczmarczyk@gmail.com) for researchers who meet the criteria for access to confidential data.

## Additional Points

*Research Highlights*. (1) Gait stability improved in most subjects after cochlear implantation. (2) Significant improvements were seen in various gait parameters, GSR, and CoM. (3) Based on CoM results, we propose a gait stability classification system.
